# Supplementary material for: Structure of tetrameric forms of the serotonin-gated 5-HT3A receptor ion channel
Source: EMBO J. 2024 Sep 4;43(20):4451–71. doi: 10.1038/s44318-024-00191-5 (PMC11480441; doi:10.1038/s44318-024-00191-5)
Supplement: Supplementary file 1 — Appendix [file 44318_2024_191_MOESM1_ESM.pdf]

Appendix for

Structure of Tetrameric Forms of the Serotonin-gated 5-HT3$_A$ Receptor Ion Channel

Bianca Introini[1,2$], Wenqiang Cui[3,4$], Xiaofeng Chu[8$], Yingyi Zhang[1,2], Ana Catharina Alves[5], Luise Eckhardt-Strelau[1], Sabrina Golusik[8], Menno Tol[5], Horst Vogel[3,5*], Shuguang Yuan[3,6*], Mikhail Kudryashev[1,2,7,8*]

1. Max Planck Institute of Biophysics, Frankfurt on Main, Germany
2. Buchmann Institute for Molecular Life Sciences (BMLS), Goethe University of Frankfurt on Main, Germany
3. The Research Center for Computer-aided Drug Discovery, Institute of Biomedicine and Biotechnology, The Shenzhen Institutes of Advanced Technology, Chinese Academy of Sciences, Shenzhen 518055, China
4. University of Chinese Academy of Sciences, Beijing 100049, China
5. Institute of Chemical Sciences and Engineering (ISIC), Ecole Polytechnique Fédérale de Lausanne (EPFL), Lausanne, Switzerland
6. AlphaMol Science Ltd, Shenzhen 518055, China
7. Institute of Medical Physics and Biophysics, Charité-Universitätsmedizin Berlin, Germany
8. In Situ Structural Biology, Max Delbruck Center for Molecular Medicine in the Helmholtz Association, In situ Structural Biology, Berlin, Germany

***Correspondence:** shuguang.yuan@cadd2drug.org, horst.vogel@stat.ac.cn and mikhail.kudryashev@mdc-berlin.de

$ indicates equal contribution.

Table of contents:

Appendix Table S1...........................2

Appendix Table S2...........................3

Appendix Table S3...........................4

Appendix Table S4...........................5

Appendix Fig. S1.............................6

Appendix Fig. S2.............................7

Appendix Fig. S3.............................8

Appendix Fig. S4.............................9

Appendix Fig. S5.............................11

References...12

Appendix Table S1: Details of data collection and model validation

	5-HT-bound		Apo		5-HT + $CaCl_2$	StA 5-HT pentamer	StA 5-HT tetramer
	Asymmetric (PDB ID: 8C21)	Symmetric (PDB ID: 8C20)	Asymmetric (PDB ID: 8C1W)	Symmetric (PDB ID: 8C1Z)	Asymmetric	Asymmetric (EMD-19420)	Asymmetric (EMD-19419)
Data collection and processing							
Microscope	Titan Krios	Titan Krios	Titan Krios	Titan Krios	Titan Krios	Titan Krios	Titan Krios
Magnification	105,000	105,000	105,000	105,000	105,000	105,000	105,000
Voltage (kV)	300	300	300	300	300	300	300
Detector	K3	K3	K3	K3	K3	K3	K3
Electron exposure (e-/Å^2)	50	50	55	55	60	186	186
Number of frames/movie	40	40	40	40	40	60 at 0 degree 10 for others	60 at 0 degree 10 for others
Defocus range (µm)	-2.1, -1.8, -1.5, -1.3 µm	-2.1, -1.8, -1.5, -1.3 µm	-2.5, -2.1, -1.8, -1.5, -1.3 µm	-2.5, -2.1, -1.8, -1.5, -1.3 µm	-2.5, -2.1, -1.7, -1.5, -1.3 µm	-3.5 ~ -5.5 µm	-3.5 ~ -5.5 µm
Pixel size (Å)	0.837	0.837	0.837	0.837	0.837	0.69	0.69
Symmetry imposed	C1	C1	C1	C1	C1	C1	C1
Number of images	11.872	11.872	7.931	7.931	12.720	309 tilt-series	309 tilt-series
Particles extracted (total)	3.510.000	3.510.000	2.843.989	2.843.989	2.633.751	30.623	30.623
Particles refined (final)	338.651	296.783	384.937	253.094	126.251	3.031	4.431
Resolution Unmasked (Å)	3.3	3.3	3.5	3.3	7.6	19.6	27.2
Resolution Masked (Å)	3.2	3.3	3.5	3.3	6.5	19.6	25.2

	5-HT-bound		Apo		5-HT + $CaCl_2$	StA 5-HT pentamer	StA 5-HT tetramer
FSC threshold	0.143	0.143	0.143	0.143	0.143	0.143	0.143
Model vs map cross-validation (Å)							
Model vs whole map	3.5	3.5	3.8	3.6	--	--	--
FSC threshold	0.5	0.5	0.5	0.5	--	--	--
Model vs. data correlation coefficients (CC)							
CC (mask)	0.77	0.78	0.73	0.74	--	--	--
CC (box)	0.65	0.61	0.63	0.64	--	--	--
CC (peaks)	0.59	0.53	0.54	0.58	--	--	--
CC (volume)	0.72	0.74	0.68	0.69	--	--	--
Mean CC for ligands	0.69	0.65	--	--	--	--	--
Validation							
Ramachandran Plot Favored (%)	94.01%	91.43 %	92.76 %	94.79 %	--	--	--
Allowed (%)	5.51 %	8.09 %	6.21 %	4.48 %	--	--	--
Disallowed (%)	0.48%	0.48 %	1.03 %	0.34 %	--	--	--
Molprobity score	1.94	2.00	1.78	1.78	--	--	--
Molprobity clashscore	10.45	9.36	5.9	7.71	--	--	--
Poor Rotamers (%)	0.07 %	0.44 %	0.29 %	0.00 %	--	--	--

Appendix Table S2: GROMACS .mdp file entries for enforced rotation MD

Parameters	ChainA	ChainB	ChainC	ChainD
rotation	Yes	Yes	Yes	Yes
rot-nstrout	1	1	1	1
rot-nstsout	10	10	10	10
rot-ngroups	4	4	4	4
rot-type0	iso	iso	iso	iso

rot-massw0	no	no	no	no
rot-vec0	-0.07<br>0.49<br>-0.87	-0.19<br>0.16<br>-0.97	0.38<br>0.29<br>0.88	0.55<br>0.12<br>0.82
rot-pivot0	7.05520 4.52350 10.27665	6.55805 3.99875 10.10755	4.15225 6.12650 9.88275	5.33165 6.69430 9.96785
rot-rate0	0.0046	0.00531	0.01891	0.00823
rot-k0	500	500	500	500
rot-fit-method0	norm	norm	norm	norm

Appendix Table S3: Time and constraint potential parameters of each step

Steps	Types	dt/fs	Time/ps	Restraint/KJmol-1 nm-2				
				Backbone	Sidechain	Ligand	Lipid	Dihedral
1	EM	1	50	4000	2000	4000	1000	1000
2	NVT	1	125	4000	2000	4000	1000	1000
3	NPT	1	125	2000	1000	2000	400	400
4	NPT	1	125	1000	500	1000	400	200
5	NPT	2	500	500	200	500	200	200
6	NPT	2	500	200	50	200	40	100
7	NPT	2	500	50	0	50	0	0
8	cMD	2	1×10^6	0	0	0	0	0

Appendix Table S4: Define the distance between the center of mass between ECD and TMD atoms

Types	CV1		CV2	
	COM1	COM2	COM1	COM2
112	1-536	914-1449	537-913	1450-1826
123	1-536, 914-1449	1828-2363	537-913,1450-1826	2364-2743
134	1-536, 914-1449, 1827-2362	2738-3263	537-913 1450-1826 2363-2737	3264-3643
224	1-536, 914-1449	1827-2362, 2738-3263	537-913 1450-1826	2363-2737, 3264-3643
145	1-536, 914-1449, 1827-2362, 2740-3275	3653-4188	537-913, 1450-1826, 2363-2739, 3276-3652	4189-4565
235	1-536, 914-1449, 1827-2362	2740-3275, 3653-4188	537-913, 1450-1826, 2363-2739	3276-3652, 4189-4565

Appendix Figures

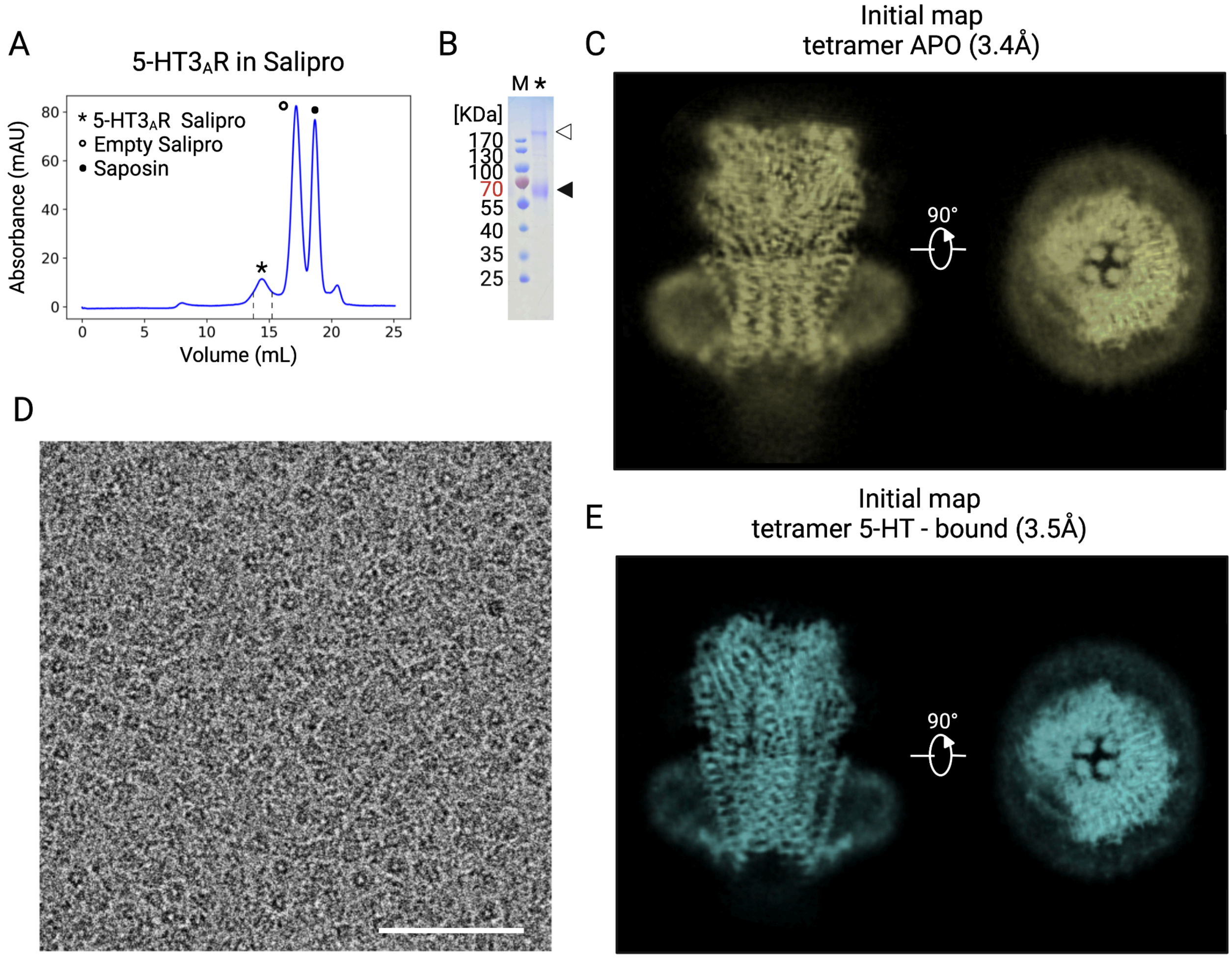

Appendix Fig. S1. Purification and isolation of the receptor.

(A) Gel filtration profile of 5HT3$_A$R reconstituted in saposins and brain polar lipids (i.e., Salipro). The asterisk indicates the peak used for further cryo-EM analysis.

(B) SDS page of 5HT3$_A$R in Salipro. The black arrowhead shows the band of the monomer (55 KDa). An empty arrowhead shows the band relative to higher oligomeric states not disrupted by the denaturing conditions.

(C and E) Cryo-EM maps of tetrameric 5-HT3$_A$R in apo state (C) and serotonin-bound (E) conformations before performing 3DVA (Punjani & Fleet, 2021). *The black background helps to visualize the maps in volume mode and appreciate the different secondary structural elements.*

(D) Representative micrograph (0.837Å/pix). Scale bar: 50 nm.

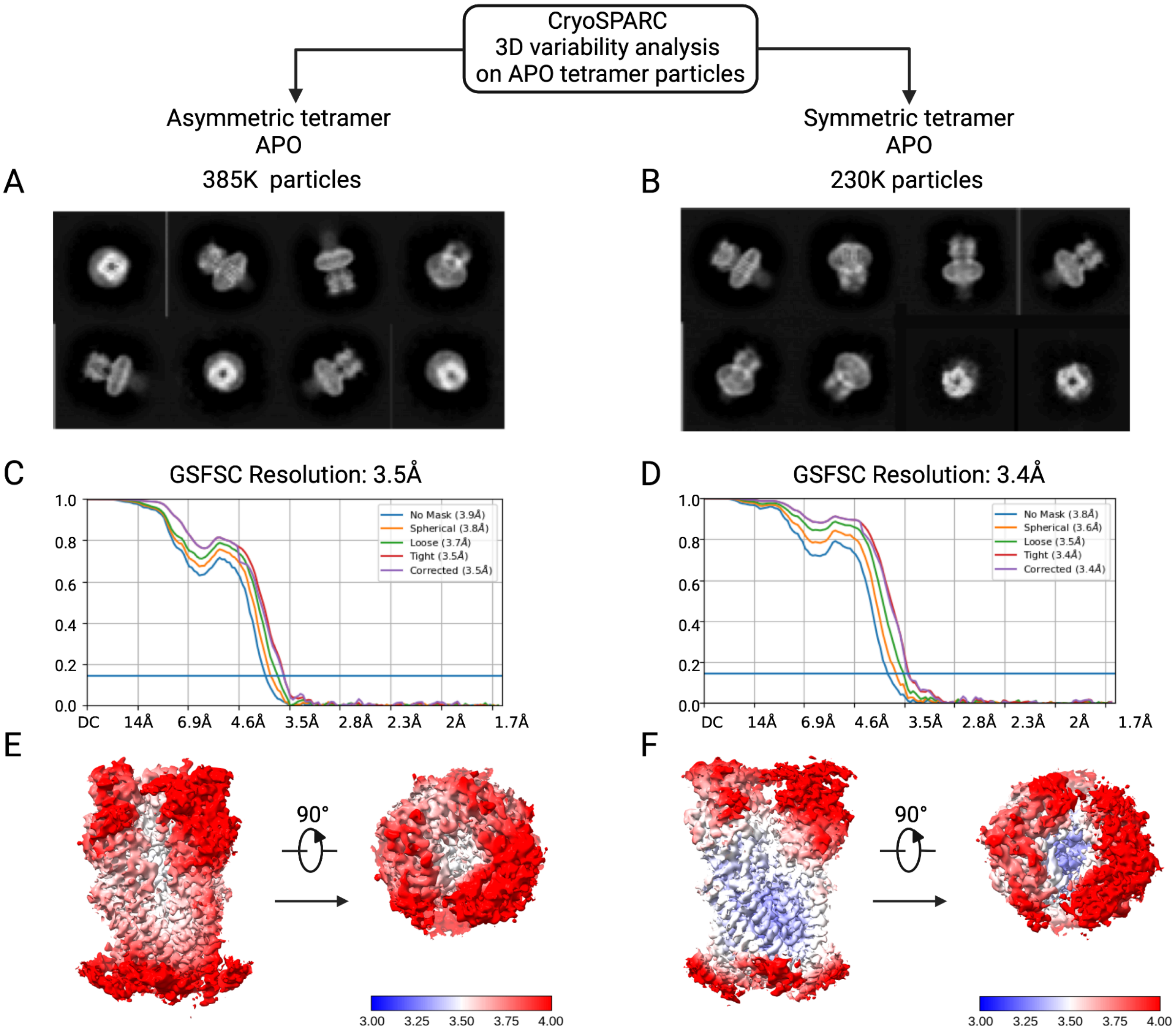

Appendix Fig. S2. Two conformations of the tetramer in apo state*.*

Each panel belongs to a particular dataset as specified at the top. (A, B) Representative 2D classes of the particles were sorted with CryoSPARC 3D variability analysis (Punjani & Fleet, 2021).

(C, D) FSC curves of the cryo-EM maps generated by the selected particles.

(E, F) Lateral and top views of the cryo-EM maps were obtained with CryoSPARC non-uniform refinement (Punjani *et al*, 2020). *Maps are colored according to the local resolution values.*

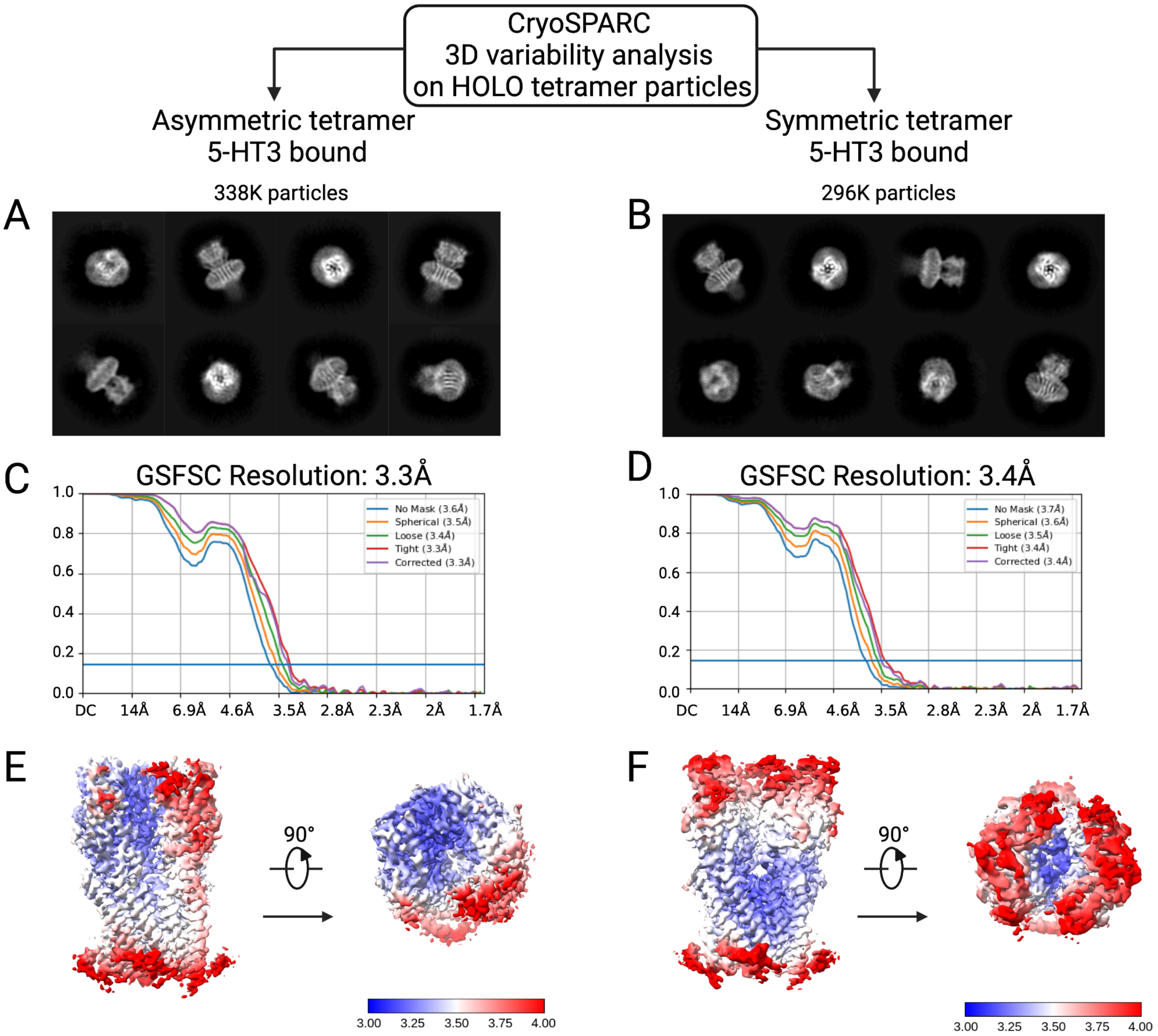

Appendix Fig. S3. Two conformations of the tetramer also in holo state*.*

Each panel belongs to a particular dataset as specified at the top. (A, B) Representative 2D classes of the particles were sorted with CryoSPARC 3D variability analysis (Punjani & Fleet, 2021).

(C, D) FSC curves of the cryo-EM maps generated by the selected particles.

(E, F) Lateral and top views of the cryo-EM maps were obtained with CryoSPARC non-uniform refinement (Punjani *et al*, 2020). *Maps are colored according to the local resolution values.*

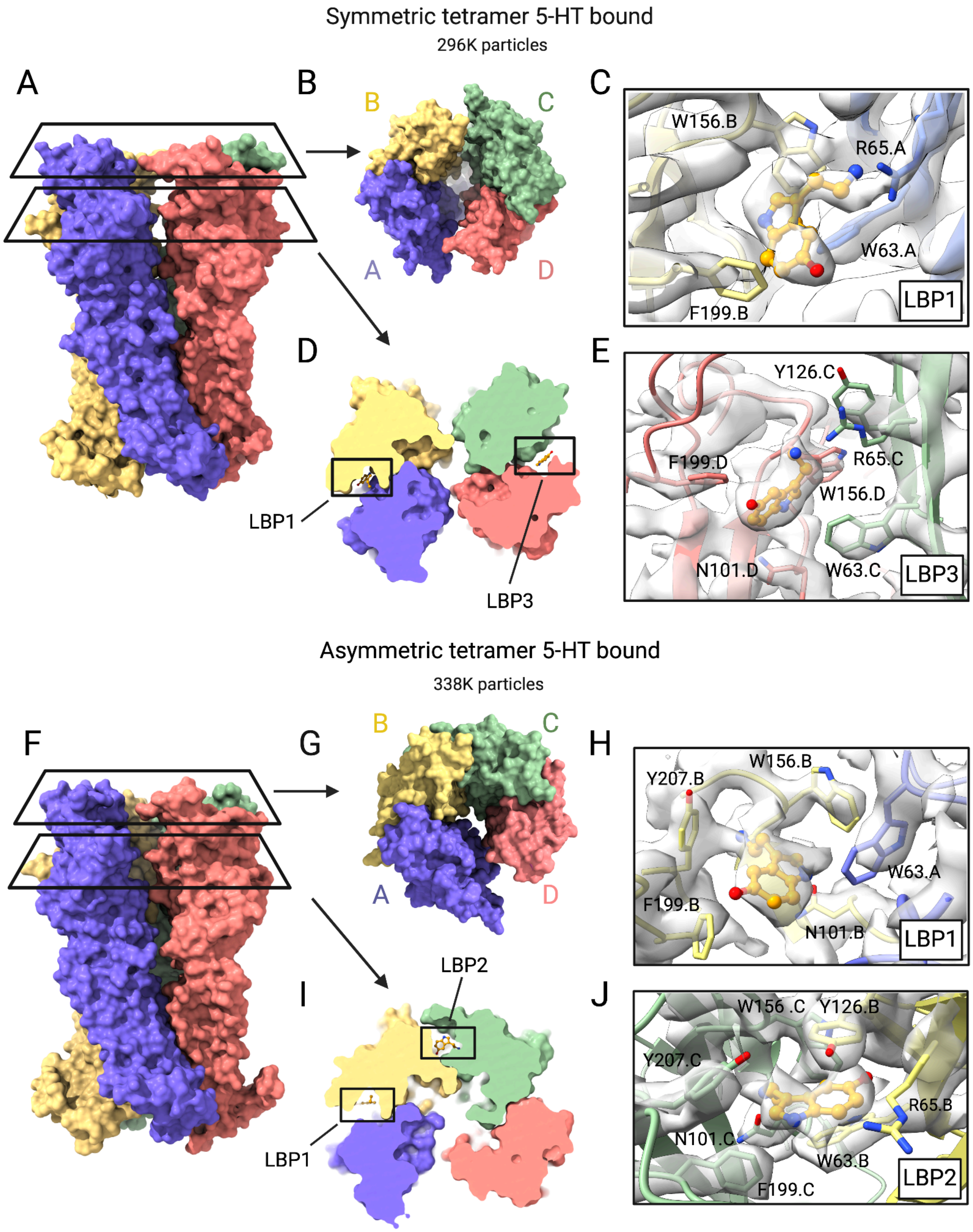
Symmetric tetramer 5-HT bound
296K particles
A
B
C
D
E
B
C
A
D
W156.B
R65.A
W63.A
F199.B
LBP1
LBP1
LBP3
Y126.C
F199.D
R65.C
W156.D
N101.D
W63.C
LBP3
Asymmetric tetramer 5-HT bound
338K particles
F
G
H
I
J
B
C
A
D
W156.B
Y207.B
W63.A
F199.B
N101.B
LBP1
LBP2
LBP1
W156 .C
Y126.B
Y207.C
R65.B
N101.C
W63.B
F199.C
LBP2

Appendix Fig. S4. Serotonin-like densities are present in the ligand binding pocket (LBP) upon the addition of the ligand.

*Models of serotonin-bound symmetric (A-E) and asymmetric (F-J) tetramers are visualized as surfaces in lateral (A and F), top (B and G)***,** *and as a section through the LBPs (D and I). For both symmetric and asymmetric tetramers the density for 5-HT was resolved in two LBP (C, D, E, and H, I, J respectively). LBPs are* ***e****numerated according to their location (i.e., the one between subunits A and B is number 1 and so on). The ligand is shown in orange as balls and sticks.*

(C, E, H, J) Close-ups of the LBPs showing serotonin in orange, models in subunit-color code, and the corresponding density maps in transparent grey (contoured at σ 0.45 for the symmetric tetramer and at σ 0.55 for the asymmetric one). (C, E, H, and J) Residues on the primary and complementary subunits are labeled with letters identifying a specific chain.

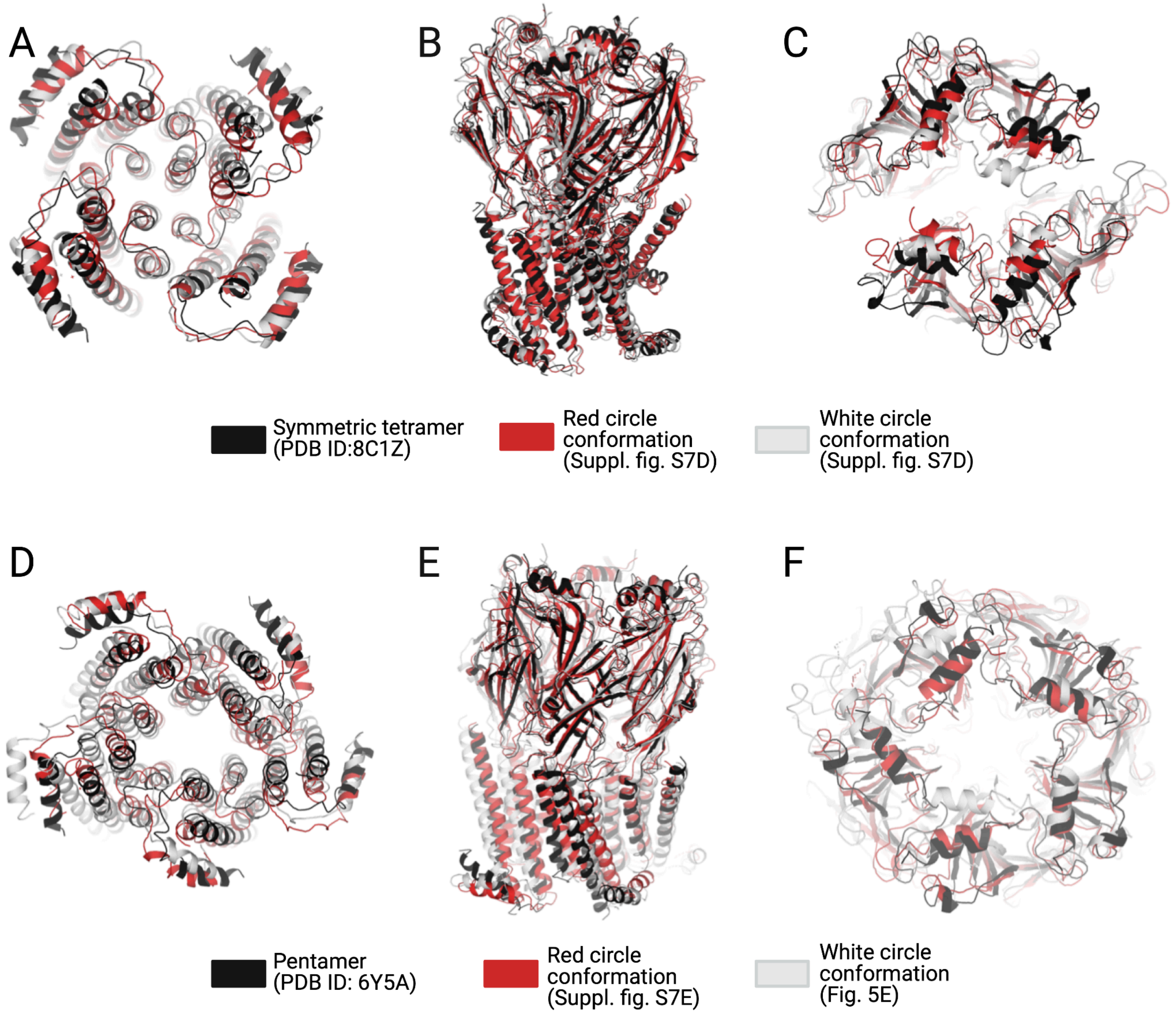

Appendix Fig. S5. Three representative conformations sampled by metaMD simulation during the formation of the symmetric tetrameter (A-C) and of the pentamer (D-F).

Each conformation corresponds and matches color with one of the energetic wells shown as circles in Fig. 5D (symmetric tetramer formation) and 5E (pentamer formation).

(A) Bottom, (B) lateral and (C) top views of three local energy minimum conformations observed during the formation of the symmetric tetramer.

(D) Bottom, (E) side and (F) top view of three local energy minimum conformations observed during the formation of the pentameric channel. For both the tetramer and the pentamer the black model shows the conformation of the cryoEM structure identified by the indicated PDB ID.

References:

Balyschew N, Yushkevich A, Mikirtumov V, Sanchez RM, Sprink T & Kudryashev M (2023) Streamlined structure determination by cryo-electron tomography and subtomogram averaging using TomoBEAR. *Nat Commun* 14: 6543

Castaño-Díez D, Kudryashev M, Arheit M & Stahlberg H (2012) Dynamo: A flexible, user-friendly development tool for subtomogram averaging of cryo-EM data in high-performance computing environments. *Spec Issue Electron Tomogr* 178: 139–151

Krissinel E & Henrick K (2007) Inference of Macromolecular Assemblies from Crystalline State. *J Mol Biol* 372: 774–797

Punjani A & Fleet DJ (2021) 3D variability analysis: Resolving continuous flexibility and discrete heterogeneity from single particle cryo-EM. *J Struct Biol* 213: 107702

Punjani A, Zhang H & Fleet DJ (2020) Non-uniform refinement: adaptive regularization improves single-particle cryo-EM reconstruction. *Nat Methods* 17: 1214–1221
