# Supplementary material for: Structure of tetrameric forms of the serotonin-gated 5-HT3A receptor ion channel
Source: EMBO J. 2024 Sep 4;43(20):4451–71. doi: 10.1038/s44318-024-00191-5 (PMC11480441; doi:10.1038/s44318-024-00191-5)
Supplement: Supplementary file 8 — Expanded View Figures [file 44318_2024_191_MOESM8_ESM.pdf]

Expanded View Figures

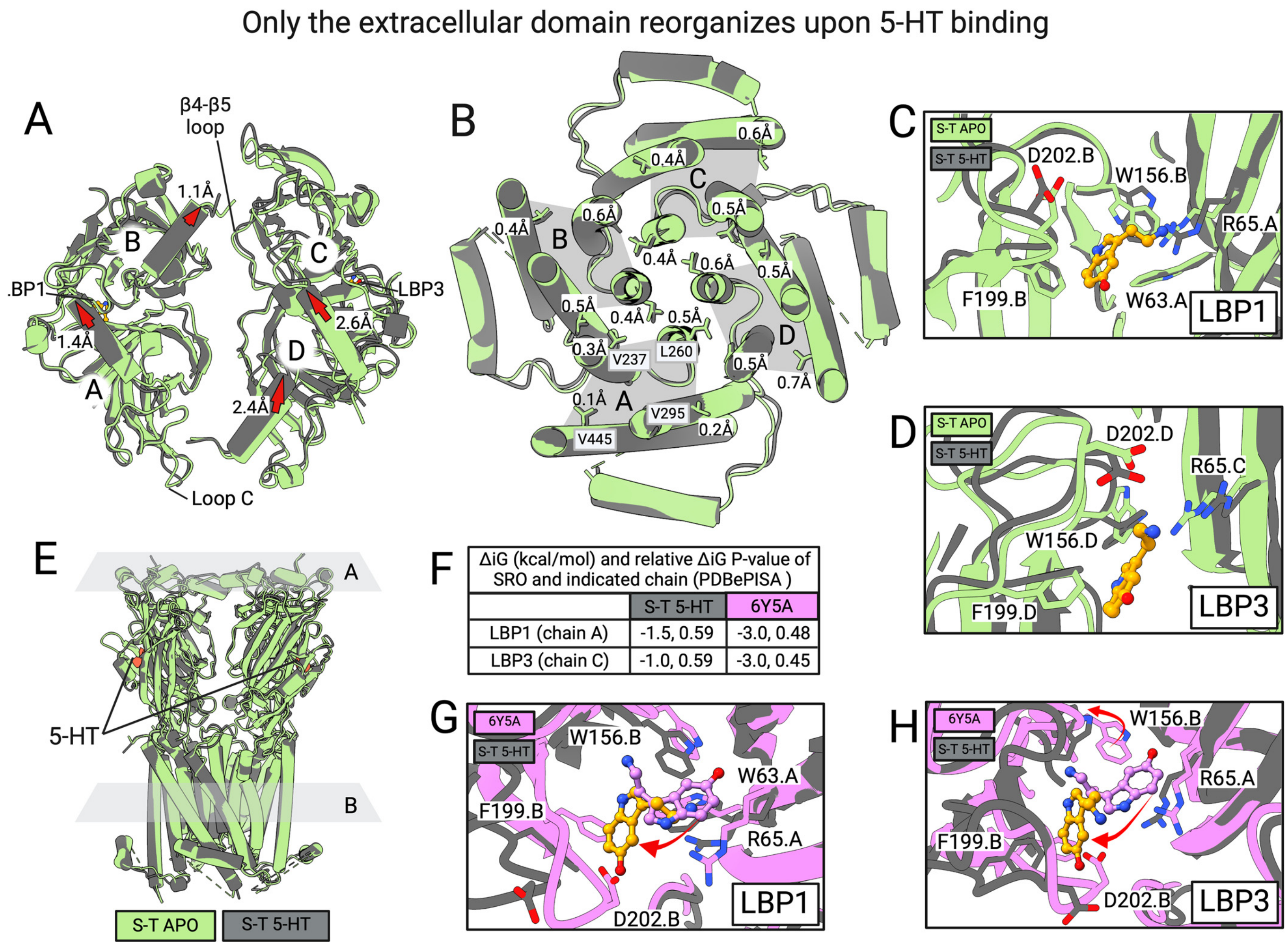

Figure EV1. The binding of serotonin does not lead to conformational changes in the transmembrane domain.

(**A**, **B**) Superposition of apo- (i.e., S-T APO) and serotonin-bound (i.e., S-T 5-HT) structures for the symmetric tetramer, with serotonin densities in dark orange. Red arrows indicate N-terminal helices movement. Serotonin densities were resolved in two LBPs for the HOLO conformation. (**B**) Cross-sections at the TMD residue L260 (9′ position) of M2 show no large movements. Displacements measured at the Cα atoms of indicated residues on each helix are shown. (**C**, **D**) Close-up of the LBPs of the apo and serotonin-bound tetramers, with serotonin in orange. (**E**) Depicts the planes shown in (**A**) and (**B**) and shows the locations of 5-HT molecules in red. (**F**) Solvation-free energy gain (ΔiG, kcal/mol) on the formation of the interface between indicated chains and ligands, calculated using PDBePISA server (Krissinel and Henrick, 2007). Negative values indicate a hydrophobic interface, with more negative values indicating stronger interaction. The ΔiG *P*-value is defined in (Krissinel, 2009). It is a measure of the specificity of the interface, *P*-values less than 0.5 suggest a specific molecular interaction. PDB IDs of each tetrameric form are reported above each panel. (**G**, **H**) Superpositions of the LBPs occupied by serotonin of the HOLO tetrameric 5-HT3_AR with the respective LBPs of the pentameric 5-HT5HT3_AR (PDB ID:6Y5A). Serotonin molecules bound to the tetramers are displayed in orange, and those bound to 6Y5A are in lavender.

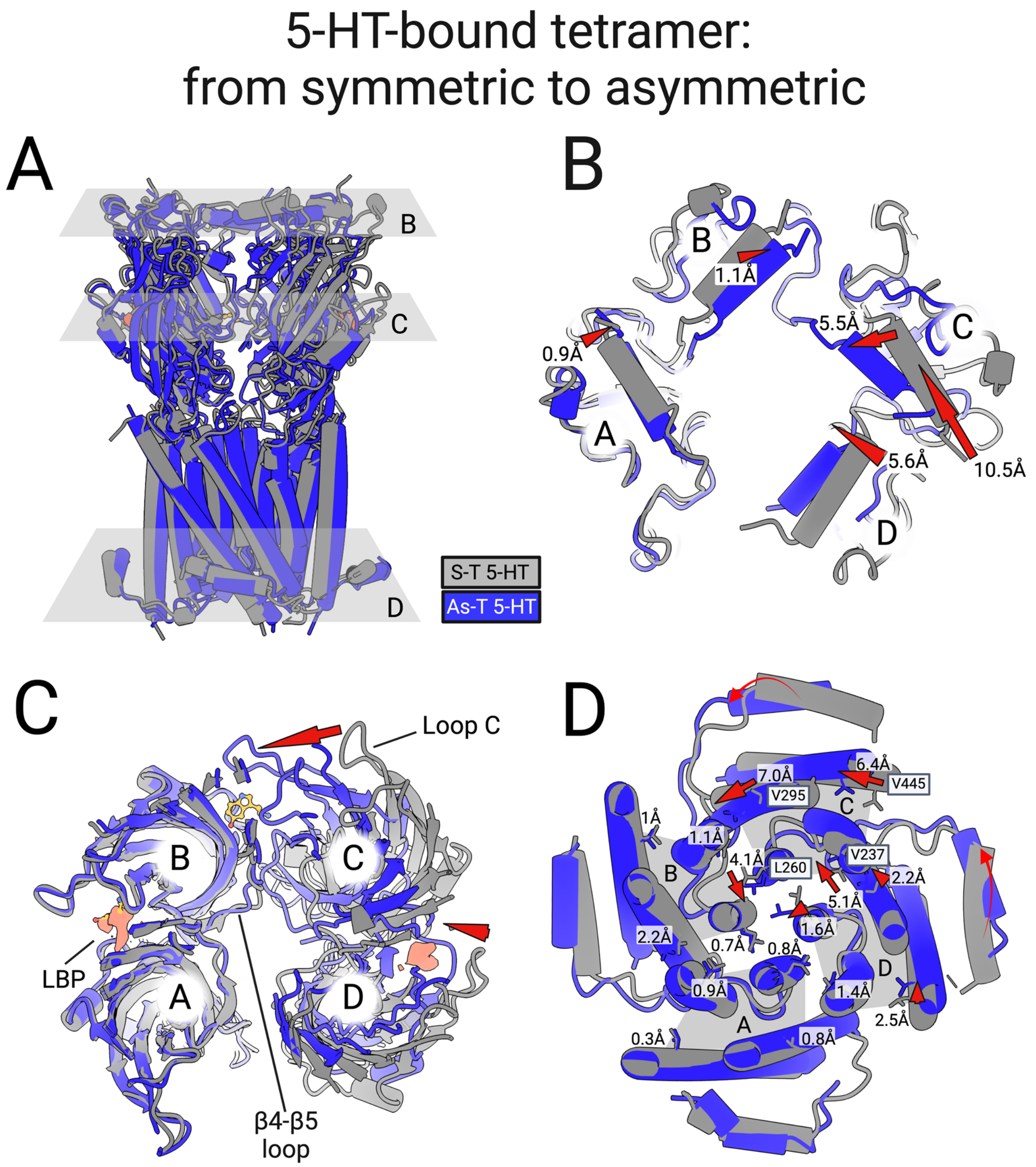

Figure EV2. The transition from symmetric to asymmetric tetramer involves the major movement of two subunits.

Superposition of the models of the holo symmetric and holo asymmetric tetramers when aligned to subunit A. (**A**) Lateral view of the indicated superposed channels with the positions of the cross-sections analyzed in (**B**), (**C**), and (**D**). The models in (**A**) are visualized as chain traces. (**B**) Top view of the superposed channels. Red arrows indicate the direction of the movement of the N-terminal helices. (**C**) Cross-section at LBPs Serotonin molecules from the symmetric holo tetramer is shown in red, while those from the asymmetric holo tetramer are in orange. (**D**) Cross-sections at the TMD residue L260 (9′ position) of M2. Displacements measured at the Cα atoms of the indicated residues on each helix in the same cross-section are shown. Also, in the presence of the ligand, both C and D helices are experiencing a downward movement while transitioning from symmetric to asymmetric.

 © The Author(s)

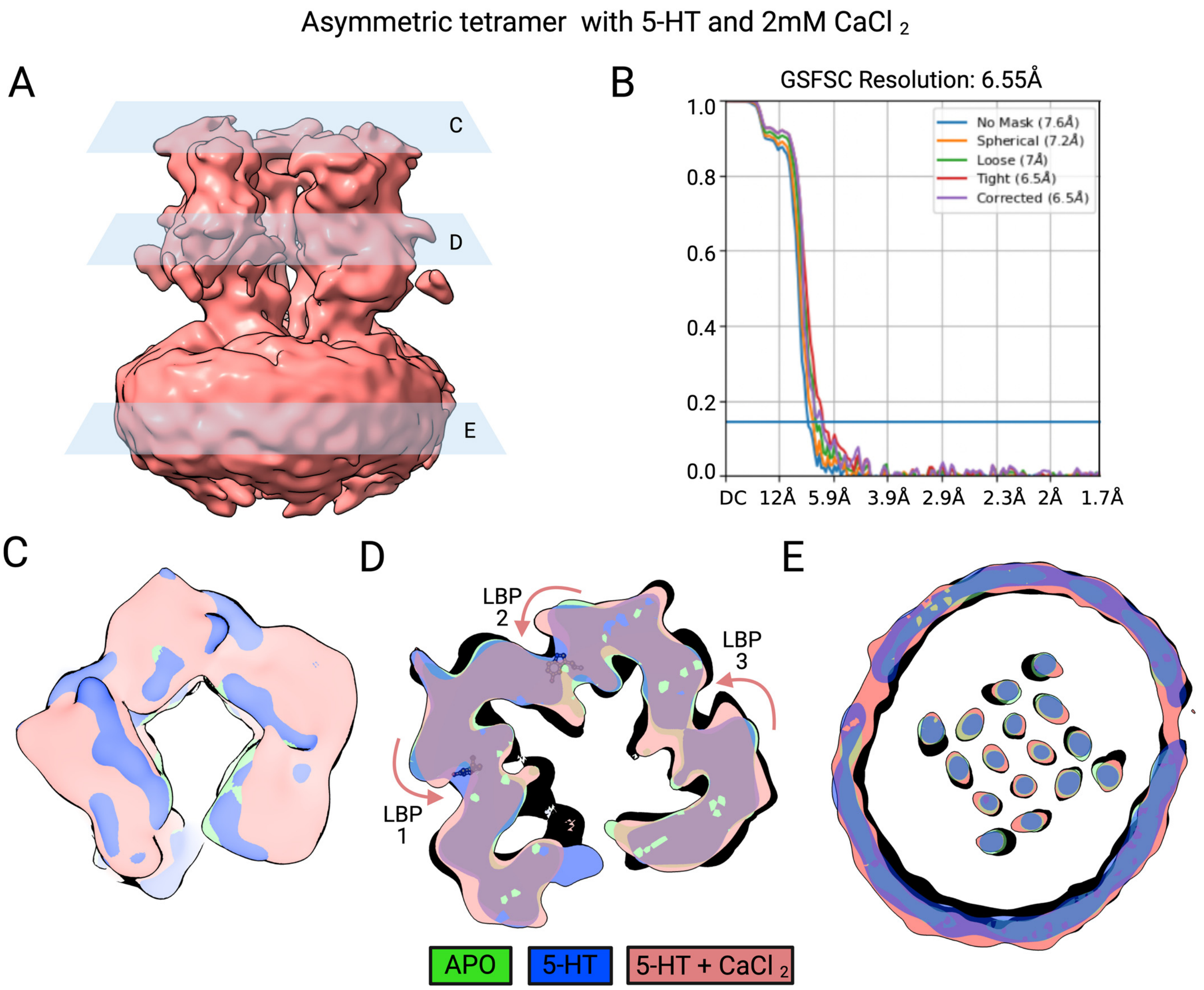

Figure EV3. The addition of $CaCl_2$ and serotonin does not cause relevant movements at the transmembrane level.

(**A**) Cryo-EM map of asymmetric 5-HT3AR tetramer in the presence of serotonin and 2 mM CaCl2 at a resolution of 6.55 Å. Positions of the cross-sections analyzed in (**C**), (**D**), and (**E**) are highlighted. (**B**) Estimating the resolution of the maps by FSC. (**C–E**) Maps of the asymmetric tetramer in the APO (green), HOLO desensitized (blue) and HOLO with $CaCl_2$ (salmon) were superposed and gaussian-filtered (standard dev 2.51) to facilitate comparison. (**C**) top view of the superposed maps. (**D**) Cross-section at the level of the ligand binding pockets (LBPs). The serotonin molecules visualized as balls and sticks are from the asymmetric holo tetramer (PDB ID: 8C21). The map suggests that in the presence of $CaCl_2$, there is a closure of the loop C of the LBP. (**E**) Cross-sections at the TMD at the level of the residue L260 (9′ position) of M2.

© The Author(s)

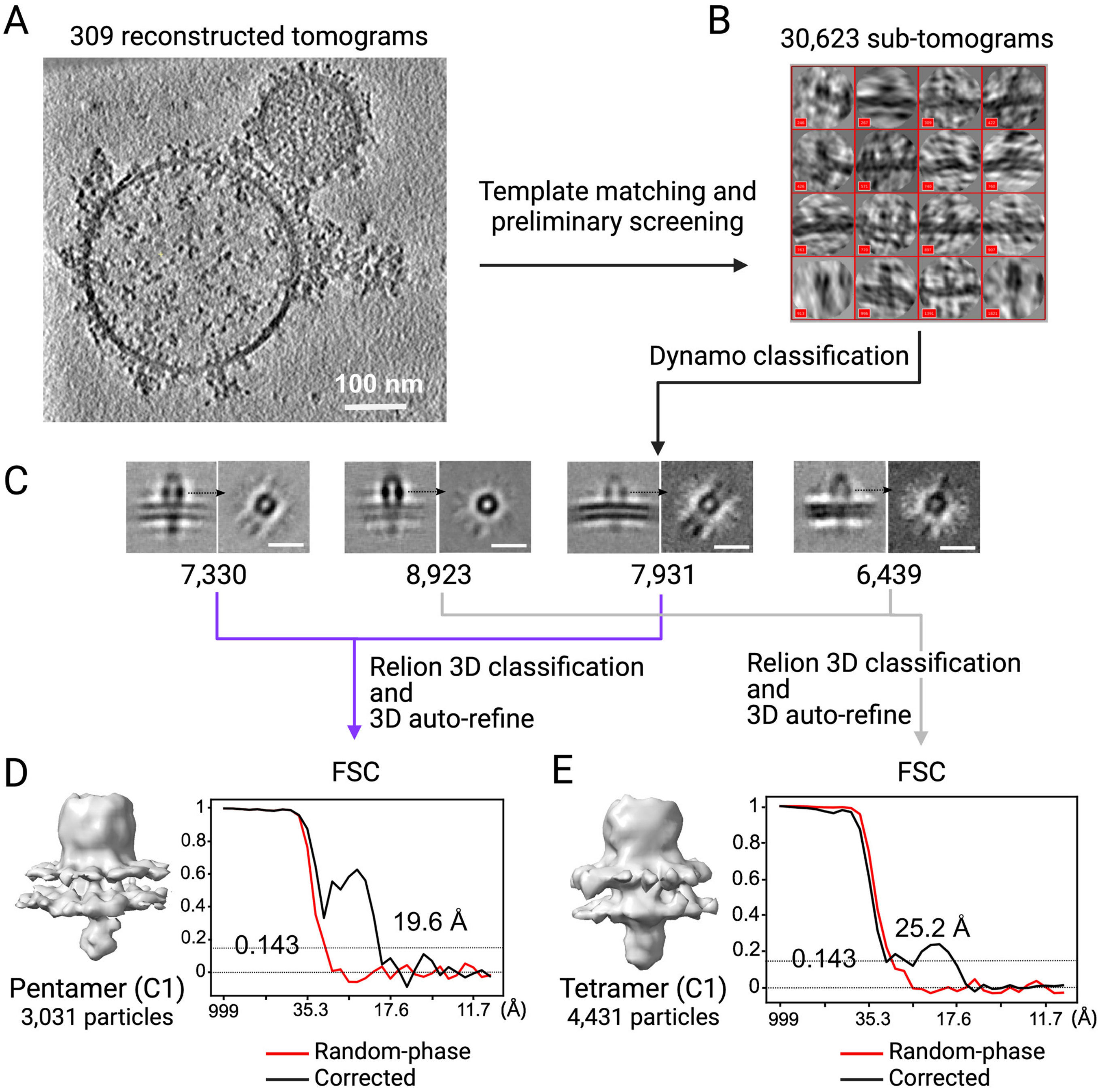

Figure EV4. Overview of the workflow of cryo-ET and subtomogram classification and averaging.

(**A**) Representative slice of a tomogram. (**B**) The particles were identified using GPU-accelerated template matching implemented in TomoBEAR (Balyschew et al, 2023). (**C**) Initially picked particles were subject to classification in Dynamo (Castaño-Díez et al, 2012). (**D**) Pentamer-looking classes were merged and processed to 19.6 Å resolution. and (**E**) the tetramer-looking classes were merged to produce a 25 Å map. No symmetry was applied.

 © The Author(s)

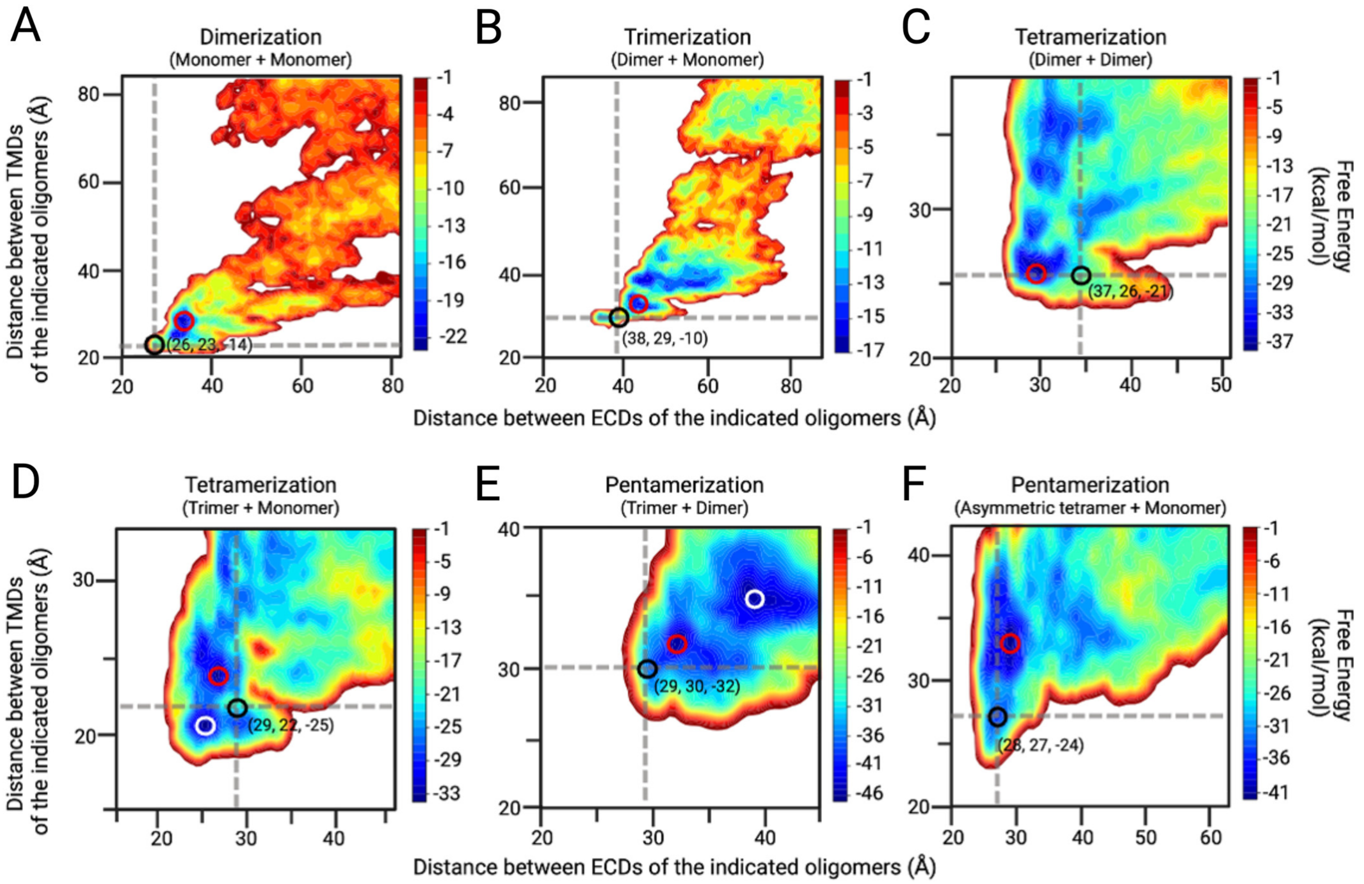

Figure EV5. Free energy surfaces (FES) acquired from metadynamics simulations for the assembly process of the $5\text{-HT}_{3A}R$.

(**A–F**) Free energy surfaces (FES) acquired from metadynamics simulations. X-axis represents the distance between the extracellular regions of the two subunits in Å, Y-axis represents the distance between the transmembrane regions of the two subunits in Å. The computed Gibbs free energy values (**G**) are expressed in kcal/mol and are represented by isoenergy lines drawn every 1 kJ/mol and shown in rainbow color. The black circle at the intersection of the two dotted lines represents the highest energy well (x, y, and z1) of the starting complex (i.e., the structure obtained from the cryo-EM experiment). The red circle represents the location of the lowest energy well (x, y, and z2) near the starting structure. A white circle represents a second low-energy well. The energy barrier is calculated as the difference between z1 and z2. The resulting value corresponds to the energy barrier (kcal/mol) that has to be overcome for the transition to a certain higher oligomeric organization. For each transition this value is depicted in Fig. 6G. A: monomer + monomer = dimer; B: dimer + monomer = trimer; C: dimer + dimer = symmetric tetramer; FD trimer + monomer = symmetric tetramer; E: trimer + dimer = pentamer; F: tetramer + monomer = pentamer.

© The Author(s)
